# Knowledge and perceptions of asthma in Zambia: a cross-sectional survey

**DOI:** 10.1186/s12890-016-0195-3

**Published:** 2016-02-12

**Authors:** Emilia Jumbe Marsden, Somwe Wa Somwe, Chishala Chabala, Joan B. Soriano, Cesar Picado Vallès, Julio Anchochea

**Affiliations:** Pendleton Family Practice, P.O. Box 38049, Lusaka, Zambia; Department of Paediatrics and Child Health, University Teaching Hospital, School of Medicine, University of Zambia, Lusaka, Zambia; Instituto de Investigación Hospital Universitario de la Princesa (IISP), Universidad Autónoma de Madrid, Madrid, Spain; Hospital Clinic, IDIBAPS, CIBERES, Universitat de Barcelona, Barcelona, Spain; Instituto de Investigación Sanitaria Princesa (IP), Hospital de la Princesa, Universidad Autónoma de Madrid, Madrid, Spain

**Keywords:** Africa, Asthma, Medications, Misconceptions, Symptoms, Zambia

## Abstract

**Background:**

Zambia is currently experiencing an epidemiological transition, from communicable to non-communicable diseases. The annual rate of physician-diagnosed asthma is estimated at 3 %. However, the general public’s knowledge of asthma symptoms and signs, and their perception of asthma remain unknown. A survey was conducted aiming to determine knowledge and perceptions of asthma among Zambians.

**Methods:**

Adults and adolescents attending four clinics in the capital, Lusaka, were surveyed using a standardized questionnaire from July 2011 to March 2012.

**Results:**

Data from 1,540 participants (mean age 30.7 years, 65 % female) were collected. Most patients (74 %) were living in low-cost housing. One hundred and sixteen (7.6 %) participants reported either a medical diagnosis of asthma or currently taking asthma medications. The most frequent asthma symptoms reported were wheezing (88 %), and waking up at night with either shortness of breath (85 %), chest tightness (85 %), or cough (67 %). Medications used to treat asthma were mostly oral short-acting beta-agonists (SABA) (59 %), inhaled SABA (30.2 %) and antibiotics (29.8 %). Inhaled steroids were only used by 16.4 % while less than 1 % were on long-acting beta-agonists (LABA). Many misconceptions were identified among the entire surveyed population with only 54.7 % believing hospitalisations are not preventable, 54.7 % believing asthma symptoms can be prevented with the right medications and 37 % believing inhalers are addictive. Nearly 60 % thought that people with asthma cannot exercise or play hard. Significantly more individuals with asthma compared to those without thought tablets are better than inhalers for the treatment of asthma (46 % vs 30 %).

**Conclusions:**

We conclude that knowledge on asthma is poor in Zambia, where there remains many misconceptions on asthma and its management.

## Background

Asthma is a serious global health problem that affects people of all backgrounds and ages. The 2010 Global Burden of Disease study estimates the current asthma burden to be greater than 334 million which is consistent with previous reports [1, 2]. Asthma prevalence within a population generally varies between 1 %–18 % for children and adults, with great heterogeneity between countries [3]. Despite the paucity of data from Africa, evidence suggests that prevalence rates have been steadily increasing over the past two decades with current rates ranging from 6–20 % in Sub-Saharan Africa [4, 5].

Zambia, a landlocked country in southern Africa with a population of 13 million is currently experiencing an epidemiological transition, from communicable to non-communicable diseases (NCD) [6]. The World Health Organisation’s (WHO) World Health Survey estimated annual rates of physician-diagnosed asthma in Zambia to be 3 %, clinical asthma to be 3 % and wheezing symptoms to be 6 % [7]. These figures may underestimate the true prevalence of asthma. In our experience treating patients in Zambia for nearly two decades, we have found that asthma tends to either be underdiagnosed or mislabelled as ‘bronchitis’, especially in children. We believe these practices have contributed to a general lack of knowledge and misconceptions about asthma, as well as stigmatisation of individuals with asthma.

In Zambia, the general population’s knowledge of asthma signs, symptoms and medication use has never been systematically investigated. Similarly, little is known about the public’s perception of asthma as a disease. In other populations, such studies have shown that parents of children with asthma and individuals suffering from asthma may have poor knowledge or misconceptions about asthma and its treatment. Common misconceptions in these studies included the idea that asthma is contagious, that it can be cured, that inhalers are either addictive or are not good treatment, that herbs play a role in asthma treatment and that asthma limits exercise [8–12]. Additionally, there were gaps in knowledge of important asthma symptoms such as shortness of breath, chest tightness and nocturnal cough [9, 12].

Poor understanding of the disease can result in under-utilisation of available health services and reduced adherence to medication [13, 14]. Ultimately this leads to poorly controlled asthma and negatively impacts quality of life.

We hypothesised that understanding of asthma in Zambia is poor and we sought to evaluate the knowledge and perceptions of asthma and the medications used to treat the disease amongst patients attending primary health care clinics.

## Methods

This was a cross-sectional survey conducted in Lusaka, Zambia, over a 9-month period, from July 2011 to March 2012. The research protocol was approved by the University of Zambia Biomedical Research Ethics Committee.

### Survey instrument

The study questionnaire was based on the Chicago Community Asthma Survey (CCAS-32), a previously validated instrument specifically designed to assess the knowledge, attitudes and perceptions of asthma among the general public [15]. Briefly, the CCAS-32 questionnaire consists of 21 dichotomous items (“true/false” or “yes/no”) and 11 Likert-scale item (“1, never true; 2, rarely true; 3, sometimes true; 4, often true; and 5, always true”). The survey captures insight into nine content domains related to asthma and its management: (1) symptoms; (2) stigma/acceptability; (3) seriousness/severity; (4) perceptions of susceptibility; (5) consequences; (6) barriers to care; (7) perceptions of quality of life; (8) treatment/utilization of health care; and (9) triggers/environmental risk. Knowledge levels are largely captured through the dichotomous items, and attitudes and perceptions are mostly captured through the Likert-scale items. Study staff administered the questionnaire verbally in the participants’ choice of language (either English or Nyanja, a local dialect which is the most widely spoken local language in Lusaka). Demographic data were collected at the time of the survey and included age, gender, education, income, occupation, area of residence and type of housing (i.e., low, medium, or high-cost housing; this item is used as a proxy for socioeconomic status by the Zambia Central Statistical Office). Knowledge and attitudes of asthma relating specifically to medication use were also assessed.

### Study sites and participants

The study took place at 4 urban, Government-managed health centres located across the city of Lusaka. Each clinic serves a catchment area ranging from 30,000 to 50,000 people. These are “first level” clinics typical of those where the majority of the urban population seeks medical care [16].

Individuals ten years of age and older who were normally cared for at the four study sites during the study period were consecutively invited to participate. Written informed consent was obtained from both the adult participants themselves and the guardians/parents of the children who participated. Exclusion criteria were refusal to give consent or inability to communicate verbally in English or Nyanja, the most widely spoken local language in Lusaka.

### Data analysis

The sample size was estimated a priori. Given that the population of Lusaka residents attending the urban clinics was 810,000 [16], we assumed that the level of asthma knowledge was 50 %. Using a margin of error of 3 % and 95 % confidence level, the minimum sample size was calculated at 1,063 respondents. Data from the questionnaire was extracted and analysed using SPSS 17 for Windows. Each categorical item from the questionnaire was summarized by frequency count. Likert-scale items were converted to true/false items as per the following: False (1 - never true; 2 - rarely true; 3 - sometimes true) and True (4 - often true; and 5 - always true). Socio-economic status and educational background were analysed as outcome variables. All data were quality controlled centrally and a homogeneous template to translate all coding was applied. Variables were then double-checked by the principal investigator, and values that were considered as potential errors or outliers were individually discussed and confirmed, or removed. Comprehensive tabulations with ranges, mean and standard deviation of all quantitative variables, and percentages of all qualitative variables, were conducted. Prevalences were presented as percentages with a 95 % confidence interval. Differences within groups were compared using Chi-squared tests for categorical variables, and Student *t* test for continuous variables. A *p*-value lower than 0.05 was considered statistically significant.

## Results

Data were collected from 1,540 participants, which represented 99 % of individuals who were invited to participate. The primary reasons for refusal to participate were mistrust of what would be done with the information gathered and concern for delay in receiving medical care. Demographic characteristics of study participants are shown in Table 1. Mean age was 30.7 years and 65 % were female. Nearly three-quarters of participants lived in low-cost housing and 8 % lived in high-cost housing. Twenty-three percent of participants reported an education level up to primary education, 55 % reported they had received secondary education, and 19 % reported higher than secondary education levels. One-hundred and sixteen (7.6 %) participants reported that they either had been diagnosed with asthma or were currently taking asthma medications. Compared with the participants without asthma, individuals with asthma (either diagnosed or self-reported) were older (mean age 37.4 years, *p* < 0.001). There were no statistical differences by gender, residential area, educational, employment or economic status.

**Table 1:** Demographic characteristics of all participants and those with asthma (either diagnosed or treated self-report)

	All *n* (%)	Diagnosed or treated asthma *n* (%)	No asthma *n* (%)	*P* value*
	1,540 (100 %)	116 (7.6 %)	1,417 (82.4 %)	
Male	538 (35.1)	39 (33.6)	499 (35.2)	0.762
Age in years, mean ± SD	30.7 ± 12.3	37.4 ± 17.4	30.2 ± 11.6	<0.001
Age interval				
10–19 years old	228 (14.9)	10 (18.6)	218 (15.4)	<0.001
20–29 years old	612 (39.9)	38 (32.8)	574 (40.5)	
30–39 years old	413 (27.0)	30 (25.9)	383 (27.0)	
40–49 years old	153 (10.0)	12 (10.3)	141 (10.0)	
50–59 years old	74 (4.8)	11 (9.5)	63 (4.4)	
60–69 years old	28 (1.8)	6 (5.2)	22 (1.6)	
70 years and older	24 (1.6)	9 (7.8)	15 (1.1)	
Residential area				
Low cost	1132 (74.0)	84 (72.4)	1048 (74.2)	0.917
Medium cost	274 (17.9)	22 (19.0)	252 (17.8)	
High cost	123 (8.0)	10 (8.6)	113 (8.0)	
Educational attainment				
No education	26 (2.6)	7 (6.1)	19 (2.1)	0.088
Some primary	104 (10.4)	11 (9.6)	93 (10.5)	
Completed primary	128 (12.8)	18 (15.7)	110 (12.4)	
Some secondary	334 (33.4)	41 (35.7)	293 (33.1)	
Completed secondary	218 (21.8)	18 (15.7)	200 (22.6)	
More than secondary	189 (18.9)	20 (17.4)	169 (19.1)	
Employment/student status				
Formally employed	218 (21.5)	29 (25.0)	189 (21.0)	0.597
Self employed	262 (25.8)	25 (21.6)	237 (26.4)	
In school/College/University	177 (17.4)	19 (16.4)	158 (17.6)	
A dependant	358 (35.3)	43 (37.1)	315 (35.0)	
Economic band (USD)				
Less than $100	123 (26.1)	16 (30.2)	107 (25.6)	0.910
$100–$199	135 (28.7)	16 (30.2)	119 (28.5)	
$200–$399	103 (21.9)	11 (20.8)	92 (22.0)	
$400–$999	90 (19.1)	8 (15.1)	82 (19.6)	
Greater than $1,000	20 (4.2)	2 (3.8)	18 (4.3)	

*All statistical comparisons were performed with Chi^2^ tests, except for age which was performed with student *t* test

Symptom frequency and treatment patterns among the 116 individuals with asthma are shown in Table 2. The most frequent asthma symptoms reported were wheezing (*n* = 102: 88 %) and waking up at night with either shortness of breath (*n* = 99: 85 %), chest tightness (*n* = 99: 85 %), or cough (*n* = 78: 67 %). There were no statistically significant differences by gender. In all, 82 % of male and 75 % of female individuals with asthma reported currently taking some form of asthma medication. Medications used to treat asthma in male and female individuals, respectively were oral short-acting beta-agonists (SABA): [71.1 % vs. 53.9 % *p* = 0.006], antihistamines (7.7 % vs. 20.8 % *p* = 0.11), oral steroids (7.7 % vs. 15.6 % *p* = 0.199), theophyllines (5.2 % vs. 22.4 % *p* = 0.06) and antibiotics (35.9 % vs. 33.8 % *p* = 0.528). Aside from inhaled SABA, other inhaled medications were used less often: SABA (41.0 % vs. 24.7 % *p* = 0.131), inhaled steroids (18.2 % vs. 10.4 %, *p* = 0.032), and long-acting beta-agonists (LABA) (2.6 % vs. 0 % *p* = 0.33). No participants reported use of inhaled LABA/steroid combination.

**Table 2:** Symptom frequency and treatment medication in asthmatics, by gender

	All (*n* = 116)	Male (*n* = 39)	Female (*n* = 77)	*P* value*
*Current symptoms*	*n* (%)	*n* (%)	*n* (%)	
Have you had wheezing or whistling in your chest at any time in the last 12 months?	102 (87.9)	35 (89.7)	67 (87.0)	0.771
Have you been at all breathless when the wheezing noise was present?	96 (82.8)	31 (88.6)	65 (97.0)	0.177
Have you had this wheezing or whistling when you did not have a cold?	44 (37.9)	13 (38.2)	31 (46.3)	0.526
Have you woken up with a feeling of tightness in your chest at any time in the last 12 months?	99 (85.3)	32 (82.1)	67 (87.0)	0.580
Have you been woken by an attack of coughing at any time in the last 12 months?	78 (67.2)	24 (61.5)	54 (70.1)	0.405
Have you been woken up by an attack of shortness of breath at any time in the last 12 months?	99 (85.3)	33 (84.6)	66 (85.7)	1.000
Have you had an attack of asthma in the last 12 months?	112 (96.6)	38 (97.4)	74 (96.1)	1.000
Are you currently taking any medicine for asthma?	89 (76.7)	32 (82.1)	57 (75.0)	0.483
*Oral medication*				
Steroids	15 (12.9)	3 (7.7)	12 (15.6)	0.199
Theophylline	19 (16.4)	2 (5.2)	17 (22.4)	0.060
SABA	68 (58.6)	27 (71.1)	41 (53.9)	0.006
Anti-histamine	19 (16.4)	3 (7.7)	16 (20.8)	0.110
Cough mixture	9 (7.8)	0 (0.0)	9 (11.7)	0.085
Antibiotics	40 (34.4)	14 (35.9)	26 (33.8)	0.528
*Inhaled medication*				
Steroids	19 (16.4)	11 (18.2)	8 (10.4)	0.032
SABA	35 (30.2)	16 (41.0)	19 (24.7)	0.131
LABA	1 (0.9)	1 (2.6)	0 (0.0)	0.330

*All statistical comparisons were performed with Chi^2^ tests

Attitudes and perceptions among study participants relating to asthma and its management are shown in Table 3. Significantly more individuals with asthma knew the signs of asthma compared to those without asthma, including knowledge that signs included shortness of breath (92.2 % vs 77.1 %), tightness in the chest (90.5 % vs 75.1 %) and wheezing after exercise (91.4 % vs 77.7 %). In addition, more individuals with asthma compared with those without knew that asthma cannot be cured (68.1 % vs 41.3 %). Just over seventy-six percent of the surveyed population understood that inhalers were good treatment for asthma. With regards to physical exercise, a substantial number of all the participants surveyed (57.6 %) thought that individuals with asthma cannot exercise or play hard, with no significant difference between those with and without asthma.

**Table 3:** Misconceptions on knowledge and perceptions about asthma in all participants and those with asthma (either diagnosed or treated self-report)

	All *n* (%)	Asthma *n* (%)	No asthma *n* (%)	*P* value*
Is shortness of breath a sign of asthma?	803 (78.8)	107 (92.2)	696 (77.1 %)	<0.001
Is tightness in the chest a sign of asthma?	783 (76.8)	105 (90.5)	678 (75.1 %)	<0.001
Are severe headaches a sign of asthma?	339 (33.3)	42 (36.2)	297 (32.9 %)	0.466
Is a cough at night a sign of asthma?	522 (51.2)	66 (56.9)	456 (50.5)	0.201
Is wheezing after exercise a sign of asthma?	808 (79.3)	106 (91.4)	702 (77.7)	<0.001
Asthma cannot be cured.	452 (44.4)	79 (68.1)	373 (41.3)	<0.001
An inhaler is a good treatment for Asthma.	778 (76.4)	84 (72.4)	694 (76.9)	0.296
People with asthma cannot exercise or play hard.	586 (57.6)	67 (57.8)	519 (57.5)	1.000
When a person with asthma is doing well they do not need to go to the doctor.	286 (28.1)	36 (31.0)	250 (27.7)	0.444
Asthma is a common reason for many school absences.	598 (58.7)	68 (58.6)	530 (58.7)	1.000
When asthma attacks stop, you don’t have asthma anymore.	240 (23.6)	16 (13.8)	224 (24.9)	0.007
You can’t have asthma as an adult without having it as a child.	291 (28.6)	17 (14.7)	274 (30.3)	<0.001
Hospitalizations for asthma are preventable.	376 (36.9)	45 (38.8)	331 (36.7)	0.085
Asthma symptoms can be prevented with the right medications.	556 (54.7)	63 (54.3)	493 (54.7)	0.141
Asthma is a serious health problem in Zambia.	614 (60.4)	83 (71.5)	531 (58.9)	0.080
Asthma care is expensive.	354 (34.8)	44 (37.9)	310 (33.8)	0.738
When a person has an asthma attack they should see a doctor immediately.	873 (85.8)	104 (89.7)	769 (85.4)	0.021
University Teaching Hospital is the best place to get treated for an asthma attack.	401 (39.5)	40 (34.5)	361 (40.1)	0.005
People can become addicted to inhalers for asthma treatment.	386 (37.0)	50 (43.1)	336 (37.1)	0.355
Tablets are better than inhalers for asthma treatment.	323 (31.8)	53 (45.7)	270 (30.0)	0.003
African doctors can cure asthma.	17 (1.7)	1 (0.9)	16 (1.7)	0.673

*All statistical comparisons were performed with Chi^2^ tests

Concerning asthma perceptions, only 36.9 % of the entire study population reported that hospitalisations for asthma were preventable and 54.7 % believed asthma symptoms could be prevented with medications. With regards to perceptions towards inhaled medication, 37 % of the study participants believed the latter were addictive, with no difference between those with and without asthma. A significant number of individuals with asthma compared with those without (45.7 vs 30.0 %) thought oral tablets were better than inhalers for asthma treatment. Overall, 60.4 % of participants agreed that asthma is a serious health problem in Zambia. Misconceptions on asthma knowledge and perceptions about the disease in those individuals with asthma (self-reported) were common (Table 4).

**Table 4:** Misconceptions on knowledge and perceptions about asthma in those with asthma (diagnosed or treated self-report), by socioeconomic status

	Low (*n* = 84)	Medium (*n* = 22)	High (*n* = 10)	*P* value*
Asthma cannot be cured	52 (61.9)	19 (86.4)	8 (80.0)	0.063
When asthma attacks stop, you don’t have asthma anymore.	14 (16.7)	1 (4.5)	1 (10.0)	0.319
You can’t have asthma as an adult without having it as a child.	16 (19.0)	1 (4.5)	0 (0.0)	0.090
Is shortness of breath a sign of asthma?	81 (96.4)	18 (81.8)	8 (80.0)	0.024
Is tightness in the chest a sign of asthma?	79 (94.0)	19 (86.4)	7 (70.0)	0.038
Is wheezing after exercise a sign of asthma?	77 (91.7)	20 (90.9)	9 (90.0)	0.981
When a person has an asthma attack they should see a doctor immediately.	74 (88.1)	22 (100.0)	8 (80.0)	0.448
University Teaching Hospital is the best place to get treated for an asthma attack.	31 (36.9)	7 (31.8)	2 (20.0)	0.545
Tablets are better than inhalers for asthma treatment.	42 (50.0)	6 (36.4)	3 (30.0)	0.302

*All statistical comparisons were performed with Chi^2^ tests

**Fig. 1:**
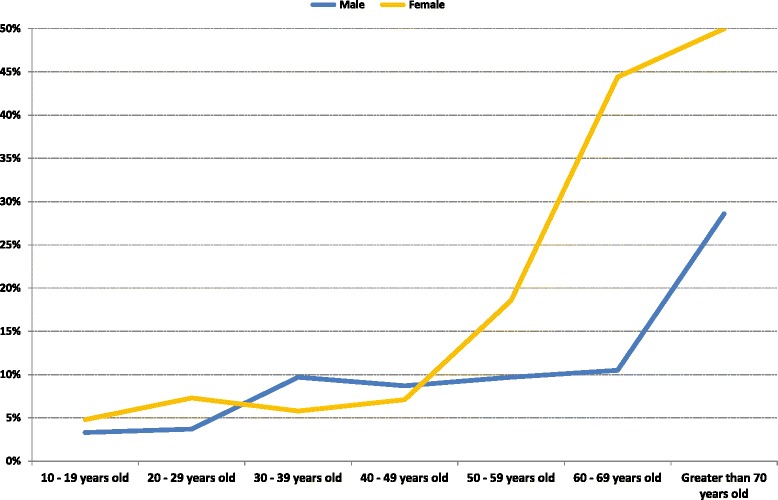
Prevalence rates of asthma (diagnosed or treated self-report) by gender and age band

Rates of health professional-diagnosed and self-reported asthma are shown in Fig. 1. The observed frequency of 7.6 % (95 % C.I. 6.2–9.0) was homogeneous from adolescence up to age 50 years, with no differences by gender. In older participants, the observed frequency of asthma increased substantially to 20.6 %, more so in women than in men (27.4 % vs 14 %).

## Discussion

This study describes, for the first time knowledge and perceptions of asthma in Zambia. We found that knowledge about asthma is generally poor and we identified misconceptions about inhaled medications that are comparable with findings from other developing countries [8, 10, 11]. Individuals with asthma were significantly more knowledgeable on asthma signs than those without, which is not surprising and is consistent with other studies [17, 18]. However, even individuals with asthma demonstrated numerous knowledge gaps and misconceptions relating to disease characteristics, asthma management and quality of life. Such attitudes can have a negative impact on patient care and quality of life [19, 20].

Misconceptions were identified regarding inhaled medications. While the majority of individuals with asthma thought inhalers are a good treatment for asthma, many asthmatics reported that inhalers are addictive and inferior in efficacy to tablets. Our finding that inhaled medications were perceived to be addictive has also been reported by others with rates ranging from 37–48 % [11, 13, 21]. This misconception could have a negative impact on adherence to medication and asthma control. It has also been implicated in the preference of treatment towards oral rather than inhaler medications [11, 22]. Indeed, our study found that most individuals with asthma were currently taking oral SABA medications, closely followed by inhaled SABA. Only a small proportion of patients were on inhaled steroids. Although antibiotics do not form part of the routine management of asthma, they were the second most administered oral medication, reflecting either a culture of over prescription by physicians or the unregulated access to ‘over the counter’ antibiotics in commercial pharmacies. It is not uncommon for antibiotics to be perceived as an important aspect of asthma treatment in developing countries [23].

International asthma guidelines state that effective management of asthma requires a self-management approach encompassing a strong partnership between the patients and the health care workers [24]. In this model, patients should be empowered to gain the knowledge, confidence, and skills to assume a major role in the management of their asthma. A self-management approach has been shown to reduce asthma morbidity. Conversely, poor self-control is likely to result if the patient has misconceptions about their asthma and inhaled medication [25].

Similarly, these guidelines also advocate the use of regular prophylactic inhaled medication to prevent symptoms of chronic asthma, and regular bronchodilator therapy as required for symptomatic relief [26]. Inhaled corticosteroids have also been shown to be effective in developing countries, reducing hospital admissions and emergency room visits by up to 80 % [27, 28]. A study in Zambia many years ago showed a reduction in asthma admissions when inhaled therapy was used [29]. Despite this evidence, up until 2013 the Zambian Standard Treatment Guidelines (STG), a set of nationally endorsed treatment guidelines covering various medical conditions used by health workers in the public health sector, emphasized the use of oral therapy as first line for mild cases of asthma with inhaled therapy reserved for acute severe exacerbations [30]. These guidelines were silent on the use of prophylactic inhaled steroids for prevention of chronic asthma symptoms.

The lack of inhaled medications in the Zambian STGs may have resulted in inconsistent availability of metered dose inhalers (MDIs) in the public health sector, and could have been a contributing factor to patients’ inclination toward oral medications for asthma management. Another reason could be failure by clinicians to prescribe MDIs due to concerns about their intermittent availability and prohibitive cost to patients [31]. Conversely, clinicians in the developing world may lack the knowledge on what medications to use for asthma treatment, commonly prescribing oral SABA and oral steroid for long-term management [32, 33]. Ultimately, even when physicians do prescribe inhaled medications, some patients may just prefer the oral route due to various negative preconceived ideas about MDIs [34].

Finally, our finding of a much higher frequency of asthma in adults older than 50 years is based on a small sample size and should be interpreted with caution. It may also be attributed to the mis-diagnosing of other conditions that present with similar symptoms to asthma such as chronic obstructive pulmonary disease (COPD) and bronchiectasis [35, 36]. Interestingly, a similar increase in asthma symptoms among the female population has recently been reported in Burkina Faso [37].

### Limitations of the current study

The asthma prevalence estimate of 7.6 % found in this study is lower than the observed prevalence rates of between 10–20 % found in other urban centres within Sub-Saharan Africa and could be an underestimate since it is based on self-reporting [5]. Misclassification of disease is a concern in Zambia where health workers often mislabel asthma as ‘bronchitis’ and could also result in under estimation of the asthma prevalence. The collection of information in English and Nyanja could have affected study results since it is challenging to accurately translate some technical terms related to asthma. It is our impression that some patients do not learn the names of the medications they are taking and this could have possibly affected their reporting of the medications they use.

### Implications and next steps

There remain major challenges in fully understanding the epidemiology of chronic airway diseases in Zambia and other African countries. Trends towards urbanisation and westernisation of African countries seem to be important contributors to the development of asthma [38]. The findings of this study including the observation that misconceptions about asthma are prevalent, could provide an evidence backed basis for the development of programmes designed to change asthma perceptions among both health workers and the community at large.

In Zambia, the national treatment guidelines for asthma have recently been updated (in 2013) and now recommend inhaled medications as the primary treatment option for individuals with asthma, but this is not yet widely practiced. Alongside this major improvement, however, oral SABAs are still recommended as an alternative treatment option. The STG update will ensure the provision of free and consistent supply of MDIs to health institutions, avoiding cost implications on the patient. The government and other stakeholders have important roles to play in assuring that appropriate education of health workers takes place so that they adhere to the new guidelines and prescribe the drugs rationally in primary health care facilities [39]. Ultimately these measures would have a positive impact on inhaler perception, use, compliance and asthma control.

## Conclusions

Knowledge about asthma is poor among Zambians and misconceptions are prevalent. Strategies are needed to increase education and awareness about the disease in order to improve disease management, reduce stigmatisation and work towards decreasing the societal burden of disease in Zambia.

## Abbreviations

CCAS-32: Chicago Community Asthma Survey-32 Questionnaire
COPD: chronic obstructive pulmonary disease
LABA: long-acting beta-agonists
MDIs: metered dose inhalers
NCD: non-communicable diseases
SABA: short-acting beta-agonists
STG: Standard Treatment Guidelines
WHO: World Health Organization
