# Erratum to: Knowledge and perceptions of asthma in Zambia: a cross-sectional survey

**DOI:** 10.1186/s12890-016-0204-6

**Published:** 2016-03-24

**Authors:** Emilia Jumbe Marsden, Somwe Wa Somwe, Chishala Chabala, Joan B. Soriano, Cesar Picado Vallès, Julio Ancochea

**Affiliations:** Pendleton Family Practice, P.O. Box 38049, Lusaka, Zambia; Department of Paediatrics and Child Health, University Teaching Hospital, School of Medicine, University of Zambia, Lusaka, Zambia; Instituto de Investigación Hospital Universitario de la Princesa (IISP), Universidad Autónoma de Madrid, Madrid, Spain; Hospital Clinic, IDIBAPS, CIBERES, Universitat de Barcelona, Barcelona, Spain

## Erratum:

The original version of this article [1], unfortunately contained some errors in the author list and author affiliations.

1. Name of one of the authors has an additional ‘h’: Julio Anchochea should read Julio Ancochea.

2. Affiliations of 3 and 4 are the same institution both authors Joan B. Soriano and Julio Anchochea should be affiliation with affiliation number 3.

### The final author list and revised affiliations should be as follows:

Emilia Jumbe Marsden1*, Somwe Wa Somwe2, Chishala Chabala2, Joan B. Soriano3, Cesar Picado Vallès4 and Julio Ancochea3.

### Affiliations:

1 Pendleton Family Practice, P.O. Box 38049, Lusaka, Zambia.

2 Department of Paediatrics and Child Health, University Teaching Hospital, School of Medicine, University of Zambia, Lusaka, Zambia.

3 Instituto de Investigación Hospital Universitario de la Princesa (IISP), Universidad Autónoma de Madrid, Madrid, Spain.

4 Hospital Clinic, IDIBAPS, CIBERES, Universitat de Barcelona, Barcelona, Spain.
